# A Medical Causerie

**Published:** 1915-03-20

**Authors:** 


### A MEDICAL CAUSERIE.

The appeal issued by Sir Alfred Keogh, as Director-General of the Army Medical Service, for further additions to the R.A.M.C. has come as somewhat of a surprise. The current report had been to the effect that the War Office had a long waiting list of volunteers far in excess of the needs of the situation. That the position is really very different from this is evident from the terms of the Director-General's letter. The need for medical men, both for home and foreign service, is described as acute, and every man who can arrange for his work to be done at home is urged to come forward as early as possible. A special request is made for general practitioners, surgeons, ophthalmologists, and radiographers, and the whole tone of the letter shows that the emergency is really a considerable one.

When to this is added the present difficulty of the hospitals in securing medical officers, it is evident that the demand for doctors has reached a very high point. And it is not easy to see how the demand can be met. Civil practice has its very urgent claims, and these cannot be neglected. At the same time, the first duty seems to be to the soldiers, and the request for further help is sure of a wide response. The occasion seems a suitable one for the corporate organisations of the profession to take action, so as to secure such an arrangement of duties as will allow men to respond to the request of the War Office without unfair prejudice to their private practices. Men with settled practices simply cannot afford to sacrifice these for an appointment which in the very nature of things can only be of temporary duration. On the other hand, the profession somehow or other must certainly supply the help which the Army authorities need. It never has failed in this respect, and it will not fail now.

It is to be hoped that the last has been heard of the notorious "umckaloabo," or "life everlasting," which figured in the Law Courts some little time ago as the basis of an advertised cure for consumption. The gentleman who turned up at the trial at an appropriate moment and swore that he was a medical graduate, and that the plant was well known in Liberia, has just been sentenced at the Central Criminal Court to six months' imprisonment for perjury. It turns out that the name he professed was that of a practitioner in Australia, and that he himself has never been on the Medical Register; on the other hand, he was able to boast three previous convictions.

Some information relative to payment of the voluntary hospitals for the reception of sick and wounded soldiers was afforded by a recent statement made in the House of Commons on behalf of the War Office. The hospitals, it was recognised, had not offered their services as a commercial transaction, but a grant-in-aid to the extent of two shillings per day per occupied bed is to be made, this sum being capable of a modest increase in any district at the discretion of the officer commanding. It is not certain that this payment is to take effect in the case of the Metropolitan hospitals, and, indeed, the various hospital authorities do not seem to have yet made up their minds unanimously. The occasion is certainly one for common action, and steps ought to be taken to secure this.

Elaborate arrangements have been made by the police, with the co-operation of the Royal Society of Medicine, to meet the medical and surgical position which would be created were bombs to be thrown on London by air-craft. The hospitals have been requested to state, in each individual instance, how many patients could be received for first-aid and how many could be treated in the wards. Full provision has been made for the prompt summoning of expert assistance, and this, quite properly, will not be limited to the official hospital staffs. It is understood also that the arrangements include a provision to dispatch a number of surgeons to any town on the south-east coast where an attack by bombs or ships might be made.

Practitioners should note that the London County Council has, by special resolution, made chicken-pox notifiable within the administrative county until June 30. Some few cases of small-pox have been notified, and there is always the risk that mild cases of this disease may be mistaken for chicken-pox. The large number of foreign refugees at present in London also helps to make the position urgent. From some recent experiences it seems that a number of the profession have not yet recognised that tuberculosis is now among the list of diseases compulsorily notifiable. Application ought to be made to the local sanitary authorities, who will supply special forms for the purpose.

Fellowship of the Royal Society is not an honour which falls with undue frequency on the medical profession. It is therefore all the more noteworthy that the latest list contains no fewer than four medical names. Of these the best known, at least outside the official world, is that of Dr. James Mackenzie, whose work on cardiac disease has brought him established fame. A further point of interest is that in these days of laboratory experts this work has been essentially clinical, and that its main part was accomplished amidst the stress of a busy general practice. In recent years Dr. Mackenzie has received the highest academic honours, but for a long time he worked with but scanty official recognition.

Great interest will be taken in the lecture on "The Septic Infection of Wounds" by Sir Almroth Wright, announced by the Royal Society of Medicine for the 30th inst. at 5 P.M. Rumour has it that the lecturer has said that no really efficient treatment capable of dealing with such wound infection as is now prevalent in the North of France can be expected in less than a thousand years or so. But we shall hear more about the whole matter on the 30th.

